# Hospital Meetings

**Published:** 1903-03-07

**Authors:** 


### HOSPITAL MEETINGS.

### THE SEAMEN'S HOSPITAL SOCIETY.

Mr. Nairne was the chairman of the annual meeting of this society at the Institute of Chartered Accountants, Moorgate Place, on Friday, February 27th. The year, he said, had been a very busy one, and the strain upon accommodation had been so great that the committee had felt bound to consider the recommendation made by the medical council to increase the resident staff by another assistant medical officer. Not only numbers, but the treatment required by modern standards had added enormously to the work; this increase was evident not only in the Dreadnought Hospital, but in the branch hospital at the Victoria and Albert Dock. The committee was engaged in a large expenditure on the enlarging of the hospital, on the school for tropical medicine, and on a new out-patients' department. The last gave rise to considerable difference of opinion among hospital administrators; many said it was not the part of hospitals to give relief to all such cases as applied, and that many ought to be left to the local authority. The hospital's policy was to help all who were making a living out of ships and shipping, and not only sailors. A large percentage were people working in the docks and their families. These were considered by the committee to be eligible claimants.

Speaking on finance, the chairman said there was a great and unexpected increase in the grant from King Edward's Fund. The value of this could not be estimated too highly, because it was a recognition of the vigorous and useful work being done in the hospital and dispensaries.

In recognition of a great deal of very clever engineering in underpinning the branch hospital free of charge, he proposed that Mr. Duckham should be made a vice-president of the School of Tropical Medicine.

Mr. Nairne stated that Sir Francis Lovell had returned from his mission to the Tropics in quest of financial support for the School of Tropical Medicine, and had obtained upwards of £8,000. This had paid off the amount due from the school to the hospital, and left a balance towards the erection of the new buildings. Sir Francis had been elected Dean of the school. An income which would enable better remuneration to be paid to the teaching staff was badly needed. Comparisons were sometimes made between the London and the Liverpool schools, and he would suggest that while the latter paid more attention to research, the former devoted itself more especially to training, although investigation was by no means neglected; for example, Beri Beri and the sleeping sickness were being investigated from London, and the society had reason to be very grateful to Mr. Craggs for his munificent endowment of a travelling scholarship for three years, and now a school prize of £50 a year. Mr. Craggs afterwards alluded to his "good fortune" in having been able to endow the travelling scholarship for three years; as an honorary secretary of King Edward's Hospital Fund, he knew the valuable work the society was doing in its various branches. He thought the work of the school ought to be brought home to the men engaged in the shipping trade of London; they ought to recognise its agency in improving the health of the people they sent out to foreign lands. It used to be looked upon as a last resource to go out to the Gold Coast, and this led to the trusting of important concerns to a much lower grade of men than would otherwise have been the case. If men could go without risk to their health, the merchants of London would get an adequate return for any money they might put into the school. These remarks were supported by Mr. Page, of the firm of J. S. Morgan and Co. Speaking as a trader, Mr. Swansea also said it would be money in the merchants' pockets to support the school, since by improved conditions of life consequent on increased knowledge of tropical disease, they would be able to keep their employees abroad for longer periods than had hitherto been possible. It was difficult to get old coasters who had been long in the tropics to recognise this principle. He regretted that the results of bringing the matter before the London Chamber of Commerce had not had better results.

### THE MIDDLESEX HOSPITAL.

With Mr. Andrew Clark as chairman, the Court of the Middlesex Hospital, on Thursday the 26th ult., went through the usual formal business of adopting the annual report, sanctioning expenditure, and receiving a number of minor reports on property, etc. Sir Arthur Watson commented on the satisfactory progress of the work of the hospital generally. Improvements, he said, were being effected as perfectly as possible, and it was contemplated that later it might be found desirable to make the temporary electrical department a permanent one.

Lord Sandhurst, after referring to the valuable part played by the chaplain, reported that the nurses' home was within measurable distance of completion, and that the staff would be considerably augmented.

The Chairman said the removal of the laundry away from the basement of the hospital was a very great boon to the patients, especially those in the wards immediately above.

The improvements referred to are clinical laboratories, gynæcological theatres, the removal of the laundry and kitchens, thus setting free the basement for cold storage and disinfecting apparatus, and the erection of the nurses' home. The electrical department is in full working order, and consists of an iron building in the yard. It is fitted up with an inner room containing two Finsen lamps for lupus, two high-frequency currents, the one of greater power being used for cancer, and there is a dark room for the use of the *X*-Rays, with patients' dressing-room attached.

### KING'S COLLEGE HOSPITAL.

The sixty-fourth annual Court of Governors of King's College Hospital was held on Thursday, the 26th ult. The proposal to remove the institution into the suburbs was not referred to. Viscount Dillon presided and moved the adoption of the report of the Committee of Management, which stated that, in spite of every effort to ensure economy, the expenditure in 1902 increased to £22,009, owing to the continuous increase in the cost of provisions and to exceptional expenditure on annual repairs and cleaning. The receipts from ordinary sources totalled £15,320, and in order to meet the excess of expenditure over income, it was necessary to utilise the whole of the legacies received during the year for the current expenses of the hospital, and also to make use of the £730 standing last year to the credit of deposit account. The special appeal for funds had met with some response, over £3,000 having been paid or promised; but owing to adverse circumstances, the committee was unable to bring the matter before the public to a sufficient extent to raise the large sum required. The committee recorded its regret at the death of the Archbishop of Canterbury, one of the most distinguished vice-presidents of the hospital; and at the loss the institution had sustained by the death of Professor John Curnow, M.D., for 28 years on the medical staff. Regret was also expressed that Professor William Rose, M.B., F.R.C.S., who for 10 years has been the senior surgeon of the hospital was resigning that position. The committee expressed high appreciation of his eminent services during the 26 years he was a member of the staff, and its gratification that his connection with the hospital will be maintained as one of its consulting surgeons; and also recommended his election as a vice-president of the hospital. Through the kindness of Dr. Whitfield, who presented the necessary apparatus, the hospital is now able to treat lupus by means of the "light" cure. This department has done much good work during the year, and numerous cases have derived great benefit from the treatment. The committee has recently been enabled by the generous gifts of a few friends of the hospital to equip a complete laboratory for the clinical pathologist. The work of this important department has hitherto been carried on at the college at some inconvenience owing to distance, and the advantage of the change is already felt. An outside emergency staircase from the nurses' dormitory floor for use in case of fire has been erected. A ward has been named in memory of the late Mr. Matthew Whiting, in recognition of his liberality to the hospital.

### QUEEN CHARLOTTE'S HOSPITAL.

The annual meeting of Governors of Queen Charlotte's Hospital took place on the 23rd ult., and was presided over by the Earl of Hardwicke.

The Chairman said it was customary to touch upon the main points of the report; the work had not diminished, and the number of patients had varied but slightly. The medical staff were to be congratulated on the satisfactory result of their work; five patients had died, but the cause of death was independent of the reason for which the patient was admitted. He thought the committee would wish him to give expression to their unqualified gratification in the receipt from King Edward's Fund of £1,000 as a special donation. The hospital had always been recognised, but this large sum was as unexpected as it was acceptable. It would be used to discharge existing liabilities, that being the purpose for which it was given.

The Mothers' Aid Society continued its valuable work, and an increasing number of patients was helped. The Bishop of London presided at the annual meeting of this society in November, and the committee cordially associated itself with all that his lordship said about it. The committee had watched with great interest the movement of many years past which had culminated in the Midwives Act. He had no hesitation in saying that this hospital had taken a leading part for many years in the training of midwives, and the measure in every respect met with its views. The new regulations, which provided for extending the period of training of midwives and monthly nurses, were working well, and the result was eminently satisfactory. The number of pupils who entered for the longer term of four and five months respectively was at least two-thirds of the whole number. He would like to draw special attention to the privileges of donors in naming beds; the gift of £1,000 enabled a donor to name a bed in perpetuity. There was, as usual, special need for support; £5,000 was required annually to carry on the work, and at present only £2,000 was reliable in annual donations and subscriptions. This naturally made the carrying out of reforms, or any accidental expenditure, very difficult.

The motion was seconded by Mr. Cavendish Bentinck, and after the transaction of formal business the meeting adjourned.

### TOTTENHAM HOSPITAL.

Mr. Cory-Wright presided over a full attendance of subscribers and donors to the Tottenham Hospital, on Tuesday (24th ult.), when the minutes of the last meeting having been duly read and confirmed, the annual report and accounts were adopted. There were a few points, the chairman said, that called for notice. The number of patients had increased, in-patients by about 60 per cent. since 1899, and out-patients by about 100 per cent. The cost per bed, given as £52 0s. 5d., was not quite correct, owing to the reckoning of each out-patient at 2s. instead of 1s. 6d. (recommended by the Hospital Sunday Fund) which reduced the average somewhat. A hospital when worked at its full capacity cost a great deal less proportionately than when it was underfilled, and to get the lowest cost per head it should always be full. Tottenham Hospital was practically full with 64 beds occupied. Receipts last year amounted to £6,786; this included, however, a special donation of £2,000 from King Edward's Hospital Fund, and this sum was earmarked for the enlargement of the building. It had therefore been invested. Apart from that donation, the increase in income over that of last year was £355. The expenditure was £5,000, an increase of £500, which was accounted for by the extra number of patients. He thought it a very satisfactory thing that the usefulness of the hospital had been extended at so little additional cost. As to the financial position, he would like to see the debt of £500, with which 1902 ended, wiped off. Would the committee share the responsibility, each guaranteeing to raise part of this sum? Otherwise he feared that next year the debt would be £1,000. The committee had decided to yield to the pressing claims of patients, take in as many as the hospital could hold, and trust to the funds coming in. Especially their thanks were due to those who organised the carnival, which brought in £504; one of the best effects of this hospital was that the poorer people were trying, with their small means, to take their share in contributing to its support. The Ladies' Association had also done more than last year. He for one would not like to belong to any hospital that had not a similar association attached to it. It had been suggested that the tradesmen of the district should combine to support a "Tottenham Tradesmen's Bed." He would like to see not only Tottenham, but Edmonton, Hackney, Enfield and other districts each with its own Tradesmen's Bed; it was an excellent idea. The Licensed Victuallers had again contributed to the hospital instead of giving Christmas boxes; the amount sent in from this source was £121. The church collections were nothing like what he thought they should be. With regard to the future of the hospital, there was a certain amount of anxiety. There was no doubt the time would come, and very shortly, when the hospital must be enlarged. Tottenham was becoming a great centre of population, and a hospital of 70 beds would not be of the slightest use; 200 at least would be wanted. Even if the building were put up, it would be a white elephant without funds to maintain it. The question would have to be faced; the extra £2,000 from King Edward's Fund was given for this purpose. "We made," the Chairman concluded, "a very strong appeal, and this is the first satisfactory answer we have had."

After some discussion, it was agreed that the districts from which out-patients came should be mentioned in the report, in order that it might be known from what quarters adequate support ought to come.

